# Draft Genome Sequence of *Bacillus subtilis* TLO3, Isolated from Olive Tree Rhizosphere Soil

**DOI:** 10.1128/MRA.00852-18

**Published:** 2018-09-20

**Authors:** Slimane Choubane, Noujoud Gabed, Omar Khelil, Ben Amar Cheba

**Affiliations:** a Département de Biotechnologie, Université des sciences et de la Technologie d'Oran Mohamed Boudiaf (USTO-MB), Oran, Algeria; b Ecole Supérieure en Sciences Biologiques d’Oran ESSBO, Oran, Algeria; c Department of Biology, College of Science, Jouf University, Sakakah, Saudi Arabia; Georgia Institute of Technology

## Abstract

In this paper, we report Bacillus subtilis TLO3, which was isolated from olive tree rhizosphere and exhibits high amylolytic activity. The genome of Bacillus subtilis TLO3 contains 4,071 protein-coding sequences carried on one chromosome (4,232,155 bp) with an average G+C content of 44.1% and 119 RNA molecules. The gene encoding α-amylase was detected, as well as other genes related to starch and cellulose hydrolysis, making the strain a potential candidate for industrial treatment of starch and lignocellulose biomasses.

## Abstract (precis)

In this paper, we report Bacillus subtilis TLO3, which was isolated from olive tree rhizosphere and exhibits high amylolytic activity. The genome of Bacillus subtilis TLO3 contains 4,071 protein-coding sequences carried on one chromosome (4,232,155 bp) with an average G+C content of 44.1% and 119 RNA molecules.

## ANNOUNCEMENT

Bacillus is a well-studied bacterial genus, and its best representative species, Bacillus subtilis, was the first Gram-positive bacterium to have its genome entirely sequenced (1). Since then, B. subtilis has become one of the best-understood prokaryotes in terms of molecular biology and cell biology because of its relatively large genome and genetic amenability (2). With the advent of next-generation sequencing, more and more complete genome sequences of B. subtilis are being reported (3–6), thus deepening our understanding of this species.

Here, we describe the newly isolated Bacillus subtilis TLO3 as a starch-degrading bacterium. The strain was isolated from samples of rhizospheric soil of an olive tree in Tlemcen, Algeria. Serial dilutions (10^−6^) were done for each soil sample, and the tubes were placed in a water bath set at 80°C for 10  min to eliminate all of the vegetative forms. Then, 100 µl of the upper phase was spread onto starch agar plates.

The isolate exhibiting the highest amylolytic activity, as determined by starch degradation and a 3,5-dinitrosalicylic acid (DNS) assay (7), was selected and purified on LB medium.

**FIG 1:**
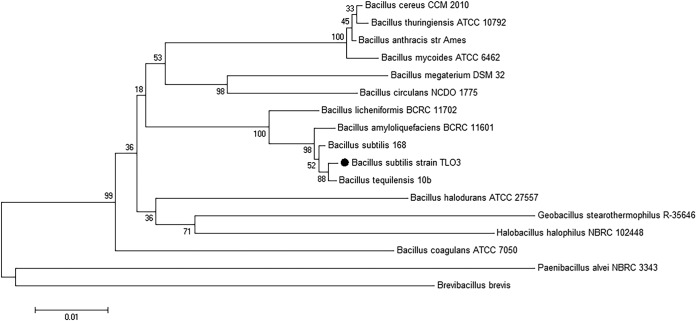
Neighbor-joining phylogenetic tree based on 16S rRNA gene sequences, showing the phylogenetic relationship between Bacillus subtilis strain TLO3 and other members of the genus Bacillus and related genera. Bootstrap values (%) for 500 repetitions are given at the nodes.

Morphological, biochemical, and physiological characterization of Bacillus species as proposed by Parry et al. in 1983 (8) was done, and results are shown in Table 1. The strain was identified using 16S rRNA gene sequencing and revealed 99% identity with Bacillus subtilis strain PVR05 (9). A neighbor-joining phylogenetic tree was constructed using MEGA 6.06 software (10) (Fig. 1).

**TABLE 1:** Morphological, biochemical, and physiological features of Bacillus subtilis TLO3

Test	Result
Gram-staining result	Positive
Cell morphology	Thin rod
Spore formation	Positive
Colony color	Cream/yellowish
Colony aspect	Rough surface, irregular edges
Biofilm	Positive
Catalase	Positive
Citrate	Positive
Lecithinase	Negative
Aerobe or anaerobe	Strict aerobe

B. subtilis TLO3 genomic DNA was extracted using a standard Gram-positive DNA extraction protocol and subjected to preparation using a Nextera XT DNA sample preparation kit (Illumina, USA). The whole-genome sequencing of the strain was done using a MiSeq (Illumina, USA) next-generation sequencer with MiSeq reagent kits version 2. Paired-end reads (2 × 300 bp) were obtained and the quality was checked by FastQC (http://www.bioinformatics.babraham.ac.uk/projects/fastqc). Total coverage of 40× was achieved and the reads (1,387,262) were assembled to reference genome B. subtilis 168 with GENEIOUS version 5.4.4 software (Biomatters, Inc.) using default parameters. A consensus sequence of 4,232,155 bp was generated from the assembled sequence and submitted to the online annotation server RAST 2.0 (11–13).

The complete genome of Bacillus subtilis TLO3 consisted of one 3,923,177-bp chromosome with 4,071 protein-coding sequences (CDS), 471 functional subsystems falling into 27 major categories, 119 RNA genes, and an average G+C content of 44.1%.

The major subsystem categories (present in order according to their number of genes) were carbohydrate metabolism, amino acids and derivatives, and cofactors, vitamins, prosthetic groups, and pigments.

The α-amylase gene was detected after genome annotation and consisted of 1,980 bp located on the genome from bp 317730 to 319709.

Genes for other starch-degrading enzymes, such as alpha-glucosidase (EC 3.2.1.20), oligo-1,6-glucosidase (EC 3.2.1.10), pullulanase (EC 3.2.1.41), and neopullulanase (EC 3.2.1.135), were detected. In addition, the strain has other glycosidases, including beta-galactosidase (EC.2.1.23), beta-glucosidase (EC 3.2.1.21), arabinofuranosidase (EC 3.2.1.55), and alpha-galactosidase (EC 3.2.1.22).

### Data availability.

The strain B. subtilis TLO3 16S rRNA sequence GenBank accession number is KR262718. The complete genome sequence was deposited in GenBank under the accession number NZ_CP021169 and the raw reads in the Sequence Read Archive under the accession number SRP158490.
